# Smart Manufacturing Processes of Low-Tortuous Structures for High-Rate Electrochemical Energy Storage Devices

**DOI:** 10.3390/mi13091534

**Published:** 2022-09-16

**Authors:** Chun-Yang Kang, Yu-Sheng Su

**Affiliations:** 1 Industry Academia Innovation School, National Yang Ming Chiao Tung University, 1001 University Road, Hsinchu 30010, Taiwan; 2 International College of Semiconductor Technology, National Yang Ming Chiao Tung University, 1001 University Road, Hsinchu 30010, Taiwan

**Keywords:** batteries, supercapacitors, template, freeze drying, magnetic field, 3D printing, laser drilling, micro manufacturing

## Abstract

To maximize the performance of energy storage systems more effectively, modern batteries/supercapacitors not only require high energy density but also need to be fully recharged within a short time or capable of high-power discharge for electric vehicles and power applications. Thus, how to improve the rate capability of batteries or supercapacitors is a very important direction of research and engineering. Making low-tortuous structures is an efficient means to boost power density without replacing materials or sacrificing energy density. In recent years, numerous manufacturing methods have been developed to prepare low-tortuous configurations for fast ion transportation, leading to impressive high-rate electrochemical performance. This review paper summarizes several smart manufacturing processes for making well-aligned 3D microstructures for batteries and supercapacitors. These techniques can also be adopted in other advanced fields that require sophisticated structural control to achieve superior properties.

## 1. Introduction

Living in the era of electric vehicles (EVs), millions of scientists, engineers, and technicians have been relentlessly improving EV battery performance. Lithium-ion batteries (LIBs) stand out among all energy storage systems for EVs because of their high energy density and great cycle life. Additionally, fast charging and discharging capabilities are critical for EVs to have a similar driving mode as that of gasoline cars [1,2,3,4,5]. Short recharge time is necessary for long-distance traveling or EV owners who do not have a private garage/parking lot equipped with a battery charger. The Vehicle Technologies Office of USDOE has announced that their program aims to reduce the EV charge time to 15 min or less [6]. Large output power is also important to have a good acceleration on a highway or when carrying heavy loads. Tesla’s superchargers can offer a very fast charge rate of 120–145 kW, shortening the recharge time to approximately 30 min [3]. Nevertheless, the threshold for recharge speed comes from the battery but not the charger.

**Figure 1:**
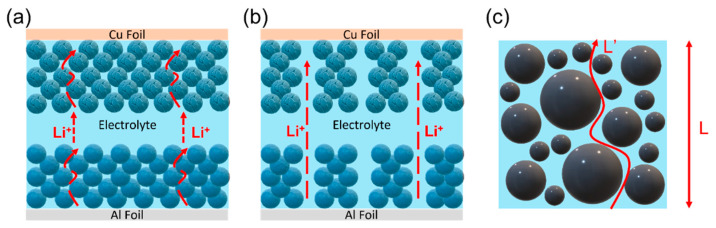
Schematic drawing illustrating lithium-ion diffusion in (**a**) the high-tortuous electrode and (**b**) the low-tortuous electrode. (**c**) Sketch of the length (*L′*) of a tortuous path in a porous electrode with a certain thickness (*L*).

When a LIB is charging, lithium ions are extracted from the cathode, migrating through the winding routes formed in the interstitial space between electrode particles. Then, lithium ions swim in the electrolyte and reach the anode surface, while the ions again have to move through tortuous channels to complete the charge transfer reactions (Figure 1a). In order to enhance the rate capability of electrochemical energy storage devices, without replacing their electrochemistry and materials, reducing the tortuosity of the electrode (Figure 1b) is an inevitable means during battery cell manufacturing. With a rational design of the electrode structure, ions can follow the shortest path to penetrate the electrode, thereby achieving fast kinetics of electrochemical reactions. Additionally, this concept may also be employed in advanced solid-state battery systems to improve their power density [7,8,9].

Figure 1c shows how tortuosity (*τ*) is defined, which is calculated using the square value of ion diffusion length (*L′*) divided by the shortest distance of the imagined ion diffusion (*L*) without considering the interference from obstacles [10]:

(1) $$\tau ={\left(\frac{L^{\prime }}{L}\right)}^{2}$$

The effective diffusivity of ions in an electrode with pores can be expressed as [10,11]:

(2) $$D_{eff}=D\left(\frac{\varepsilon }{\tau }\right)$$

where *ε* is the porosity of the electrode and *D* is the diffusion coefficient of ions in the electrochemical system. This equation clearly suggests that higher porosity and lower tortuosity will promote fast ion diffusion, enabling excellent extreme fast charging (XFC) capability for an energy storage system. In fact, the tortuosity can be expressed as a function or porosity, according to the Bruggeman relationship [12]:

(3) $$\tau =\varepsilon^{1-\alpha }$$

where *α* is the constant called the Bruggeman exponent, which depends upon the morphology, porosity, material, and particle size distribution of the components in the electrode. The major tool to determine the tortuosity of the electrode is electrochemical impedance spectroscopy (EIS) [11,13,14,15]. Some researchers have applied 2D/3D microstructure and tomographic image processing methods to give an estimate of tortuosity [10,16,17].

In this review, smart manufacturing technologies used to fabricate low-tortuous electrodes are introduced and categorized. Their correlated high-rate performance data, especially fast charging features, are also presented. For a scalable production, the processing complexity and cost-effectiveness of selected tools are taken into account when judging the practicality of the manufacturing methods and their capability to be commercialized. In the following sections, different manufacturing methods of making low-tortuous electrodes are introduced and compared in terms of electrochemical performance and commercial practicality.

## 2. Template-Directed Manufacturing for Low-Tortuous Structures

### 2.1. Anodic Aluminium Oxide Templated Methods

**Figure 2:**
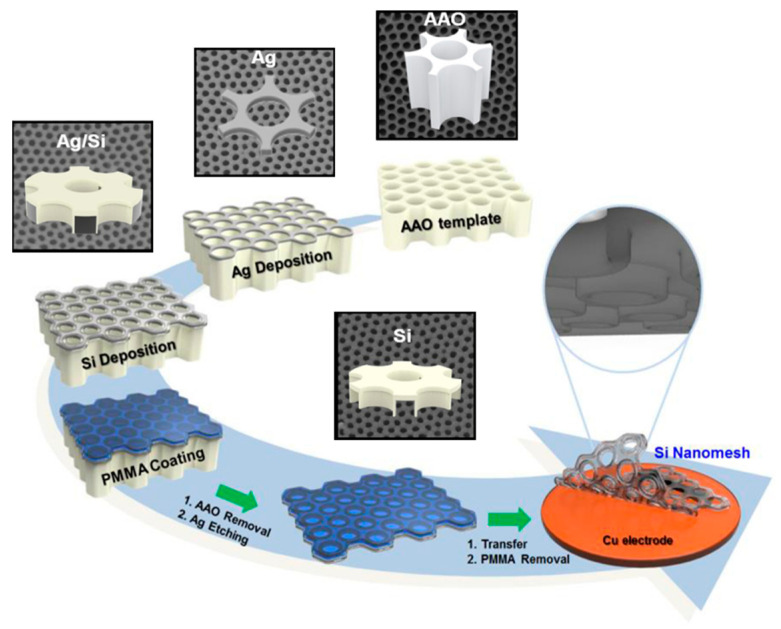
AAO-directed low-tortuous Si nanomesh electrode. Reproduced with permission from ref. [32]. Copyright 2017, Elsevier.

Using a porous template with a vertically aligned channel structure is a facile means to make low-tortuous electrodes with great power density. Porous-type anodic aluminum oxides (AAOs), made from the anodization of aluminum in acidic electrolytes, have been widely studied and utilized in energy applications [18,19,20]. AAO-derived low-tortuous electrodes possess a neatly arranged three-dimensional porous structure, which can direct ions to flow through the shortest path, thereby resulting in excellent rate capability in batteries and supercapacitors [21,22,23,24,25,26,27,28,29,30,31,32]. Figure 2 gives an example of a Si nanomesh anode for LIBs, fabricated by using AAO as the template to sequentially deposit a silver buffer layer and silicon via sputtering, followed by the coating of poly(methyl methacrylate) (PMMA) supporting the polymer layer for the electrode transfer step [32]. AAO, silver, and PMMA were all removed to complete the low-tortuous electrode manufacturing. The as-prepared Si nanomesh electrode possesses well-aligned holes ~70 nm in diameter with a density of ~96 holes μm^−2^, showing a much better rate performance than that of the planar Si nanofilm electrode [32]. The advantages of using AAO as a hard template to make porous electrodes are the (1) highly oriented channel structure, (2) low-cost template material, and (3) easy removal of template for streamlining processing. However, some delicate structural designs require the vacuum deposition of active and buffer materials, which raises the manufacturing cost and, therefore, reduces their scalability.

### 2.2. Bio-Derived Templated Methods

**Figure 3:**
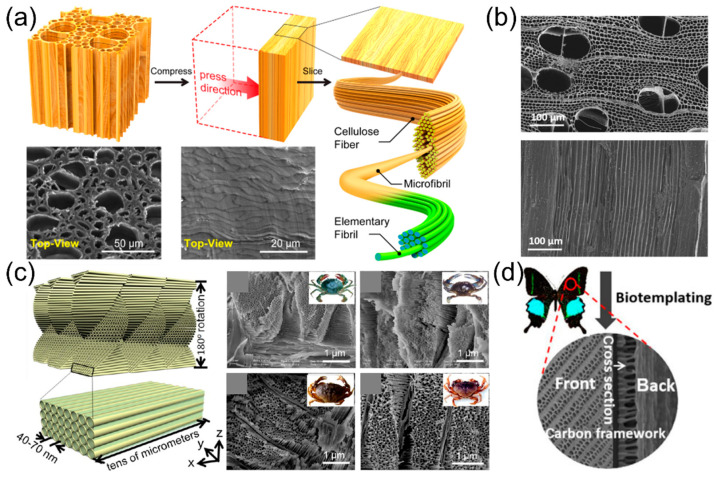
(**a**) Wood-derived aligned template structure for solid-state electrolytes. Reproduced with permission from ref. [33]. Copyright 2019, ACS Publications. (**b**) SEM images of carbonized wood slabs. Reproduced with permission from ref. [34]. Copyright 2016, Wiley-VCH. (**c**) Crab shell porous structures. Reproduced with permission from ref. [46]. Copyright 2013, ACS Publications. (**d**) Butterfly wing-derived carbon framework for supercapacitors and Na-ion batteries. Reproduced with permission from ref. [44]. Copyright 2021, Springer.

To make the templated production process more sustainable and eco-friendlier, plenty of approaches utilizing bio-derived templates have been reported. Woods [33,34,35,36,37,38,39,40,41], plant fibers [42,43], butterfly wings [44,45], and crab shells [46] were adopted as bio-templates to build low-tortuous structures for smooth ion transportation. Hu et al. developed a multiscale aligned garnet framework enabled by the wood template along with a poly(ethylene oxide) (PEO) polymer electrolyte impregnated into the mesopores, showing a high ionic conductivity (1.8 × 10^−4^ S cm^−1^) [33]. Figure 3a shows the process flowchart of the wood template preparation. First, the wood was cut perpendicular to the longitudinal direction, followed by mechanical pressing to densify the pore structure shown in the scanning electron microscope (SEM) images in Figure 3a. The fibrous nanostructure is highly aligned and can absorb abundant precursors due to the hydrophilicity of wood cellulose and capillary force induced by the orderly aligned pores [33]. The wood template can be removed after a simple pyrolysis treatment. Figure 3b shows the structure of low-tortuous and mesoporous wood-derived carbon as an anode for Na-ion batteries. The anisotropic pores have two sizes (10–15 μm from cellulose fibers and 100–200 μm from vessels) [47,48] with a wall thickness of 1–2 μm and a channel width of 10–15 μm, which enable an ultra-thick wood-derived carbon anode (850 μm) with large mass loading (55 mg cm^−2^) to deliver a high areal capacity of 13.6 mAh cm^−2^ [34]. The vertically aligned porous structure can facilitate Na-ion transport, so a much thicker electrode can be designed to achieve higher areal energy density. Crab shells can also serve as a functional template to make nanostructured battery electrodes. Cui et al. used animal shells from various crab species to create the hollow nanochannel structure with an inner diameter of 40–70 nm for the accommodation of silicon anode or sulfur cathode materials [46]. Both bio-templated electrodes demonstrated good rate capability at up to 1C and stable cyclability [46]. Butterfly wings can be used to generate 3D carbon frameworks as well for supplying channels to better access electrolytes, increase redox-active sites, and provide conductive pathways for electrons and ions in the electrode [44]. The butterfly wing-derived carbon can be adopted as both the cathode-supporting substrate and the anode active material, delivering a high energy density of 42.9 Wh kg^−1^ at a power density of 800 W kg^−1^ in supercapacitors, indicating its superior high-rate performance from the 3D hierarchical porous structure [44]. Bio-templates have several benefits such as low-cost, environmentally friendly, adjustable morphologies, and facile separation, but the supply of biomaterials must be sustainably consistent to guarantee the production quality.

### 2.3. Bubble Templated Methods

Other than hard templates, gaseous bubbles can be implemented to make 3D foams for energy storage applications. Dynamic hydrogen bubble template (DHBT) electrodeposition is one example of using bubbles to make the metallic porous structure as the electrode support [49,50,51]. The as-prepared Ni foam scaffold can be used as the 3D current collector to enhance electrical and ionic conductivity. Another approach is introducing NH_4_HCO_3_ as a foaming agent to generate NH_3_ and CO_2_ gases during the drying step of electrode preparation [52]. This oriented porous electrode structure can promote both the rate performance (≈7 times higher capacity at 5C) and mass loading (≈50% higher with a similar specific capacity), outperforming those of the conventional electrode [52]. Another group utilized NaHCO_3_, which also produces CO_2_ gas but requires washing to remove the byproduct NaOH, to form interconnected pores in the electrode [53]. The 3D porous network enabled the LiFePO_4_ cathode to have excellent rate performance, showing 35% capacity retention at 60C (charge/discharge within 1 min) [53]. Due to the high mobility of bubbles, obtaining well-aligned low-tortuous structures could be challenging.

### 2.4. Templated Phase Inversion Methods

**Figure 4:**
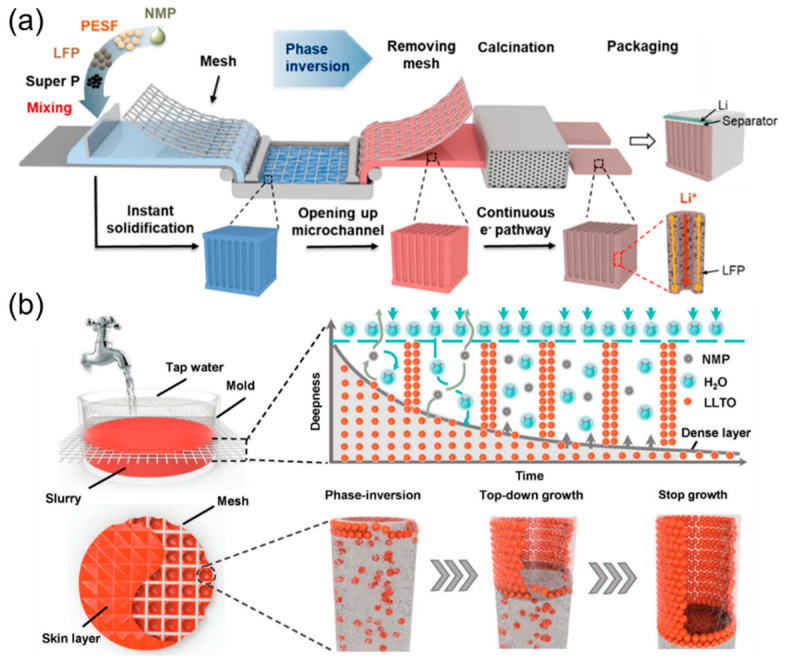
(**a**) A bilayer LFP electrode fabricated by the templated phase inversion method. Reproduced with permission from ref. [54]. Copyright 2021, ACS Publications. (**b**) LLTO solid electrolyte with vertically aligned microchannels made by the templated phase inversion method. Reproduced with permission from ref. [57]. Copyright 2018, Wiley-VCH.

The template method can also be combined with the phase inversion approach to make well-aligned structures for energy storage devices. Yu et al. covered a stainless-steel mesh on the electrode slurry when executing N-methyl-2-pyrrolidone (NMP)-based tape casting (Figure 4a) [54], and the electrode coating layer instantly solidified after water was poured on top of the slurry. The water molecules can substitute the solvent molecules to generate pores with a polymer-poor phase [55,56]. This method successfully creates not only open and aligned microchannels but also thick electrodes with high areal loading (up to 100 mg cm^−2^), resulting in both high power density and high energy density [54]. Additionally, the solid-state electrolyte system can also apply the templated phase inversion method to build 3D vertically aligned microchannels for the enhancement of ion transport. Figure 4b shows the synthetic process of the low-tortuous perovskite Li_0.34_La_0.51_TiO_3_ (LLTO) solid-state electrolyte [57]. Nylon mesh was implemented as the template with a similar phase inversion reaction between water and NMP to form a dense skin layer, and the top-down growth continued until the formation of the bottom layer was completed [57]. The LLTO electrolyte with 3D microchannels showed significantly lower interfacial resistance than that of planar LLTO (reduced from 853 to 133 Ω cm^2^) due to the shorter diffusion distance in the interdigitated structure built by the low-tortuous LLTO electrolyte and LFP cathode, demonstrating that the unique structure can support efficient and fast ion transport in the cell [57]. Other related examples including the carbon-sulfur composite cathodes [58], all-ceramic lithium-ion batteries [59], nitrogen-doped carbon electrodes for supercapacitors, and cathode support of solid oxide electrolysis cells [60] all show superior performance under high current rates by adopting the templated phase inversion method.

## 3. Low-Tortuous Structural Design via Cooling or Heating

### 3.1. Directional Freezing Methods

**Figure 5:**
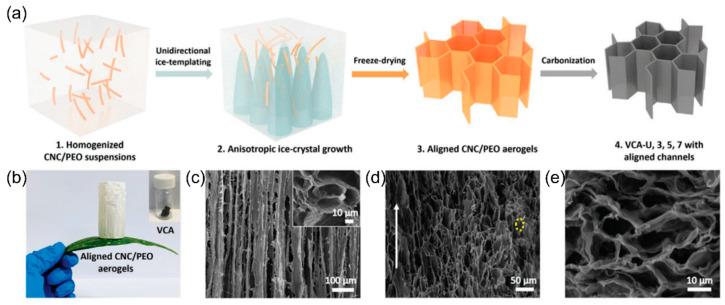
(**a**) Vertically aligned carbon aerogels synthesized by freeze drying. (**b**) The ultralight aligned cellulose nanocrystal (CNC)/polyethylene oxide (PEO) aerogels. (**c**) SEM images of cross-sectional and top view (inset) of the CNC/PEO aerogels. (**d**) Longitudinal view and (**e**) top view of the vertically aligned carbon aerogels. Reproduced with permission from ref. [61]. Copyright 2022, Wiley-VCH.

Freeze drying with controlled cooling is a famous method of introducing a low-tortuous structure into electrodes. The electrode components or precursors are first dispersed in the aqueous solution, followed by the freezing from the bottom of the solution to grow ice crystals along with the temperature gradient vertically. The unfrozen solid substances are separated by the ice columns during continuous freezing, resulting in the well-aligned channels surrounded by the electrode material/substrate. Figure 5a,b display ice-templated carbon aerogels made via the unidirectional freezing as the anode for Na-ion and K-ion batteries [61]. Figure 5c,d show the microstructure of the long and vertically aligned tubular channels formed by the removal of the ice template in the aerogel before and after carbonization, indicating that smooth and fast ion transport can be guaranteed. The honeycomb structure observed from the top view (Figure 5e) indicates that there are abundant connected electron pathways between the microchannels to ensure low electrical resistance during the electrochemical reactions. The as-made carbonaceous electrode achieved high energy densities of ≈220 and ≈118 Wh kg^−1^ and high rate performances of 206 and 148 mAh g^−1^ at 2C for Na-ion and K-ion batteries, respectively [61]. In fact, the freezing rate plays an important role to control the dimension of channel/pore formation, and both channel wall thickness and pore spacing are decreased with increasing freezing speed, leading to a higher tortuosity [62]. The directional freezing methods of building low-tortuous structures can also be applied to various material systems for high-rate batteries such as intercalation and conversion cathodes [62,63,64,65,66,67,68,69,70,71,72,73,74], scaffolds for sulfur cathodes [75,76], intercalation and conversion anodes [71,72,77,78,79,80,81,82,83,84,85], metallic lithium anode hosts [75,86], solid-state electrolytes [87,88,89,90,91], supercapacitor electrodes [39,92], and Al-ion batteries [93], showing their all-around applications.

### 3.2. Evaporation-Induced Methods

**Figure 6:**
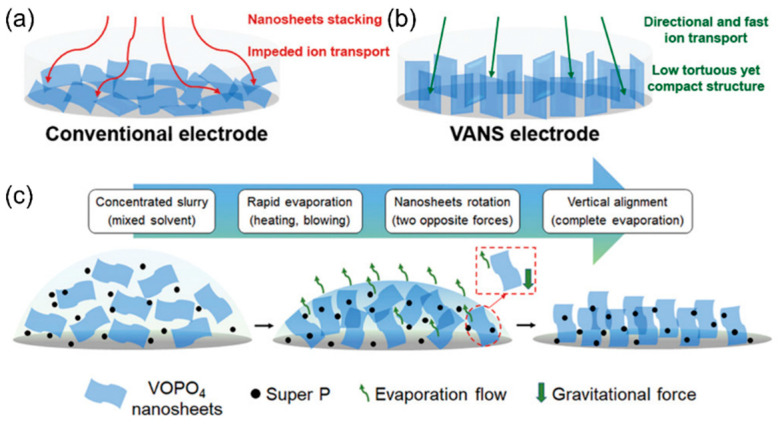
Comparison of the (**a**) conventional electrode and (**b**) VANS electrode with vertical alignment. (**c**) The evaporation-induced method of making vertically aligned electrodes. Reproduced with permission from ref. [94]. Copyright 2020, Wiley-VCH.

Opposite to the freezing methods, applying heat to the electrode may also facilitate the formation of the low-tortuous structure. Yu et al. developed a vertically aligned nanosheets (VANS) electrode that can promote directional and fast ion transport, illustrated in Figure 6a,b [94]. The evaporation-induced procedure includes slurry preparation with mixed solvents, rapid evaporation, and nanosheet rotation to obtain vertical alignment shown in Figure 6c [94]. The control of the evaporation process is key to the success of electrode orientational ordering, requiring a downward gravity force and upward evaporation flow force to offer sufficient torque for the rotation of nanosheets [94]. As a result, the electrode structure maintains upright alignment due to the fast removal of solvents, leaving no relaxation time for the nanosheets to lie horizontally again. Another study implemented evaporation-induced self-assembly to synthesize mesoporous titanium dioxide/carbon composite electrodes [95]. Multiple polymers were selected to form a suitable block copolymer paired with the titania precursor, and the internal repulsion of specific polymer blocks led to microphase separation during the evaporation process, where oriented mesopores were generated [95]. The prepared electrode can enable high areal mass loading, high bulk density, and high volumetric capacity, originated from the electrode structure with ordered pores.

## 4. Magnetic/Electric/Shear-Field-Assisted Manufacturing for Low-Tortuous Electrodes

### 4.1. Magnetic-Field-Assisted Methods

**Figure 7:**
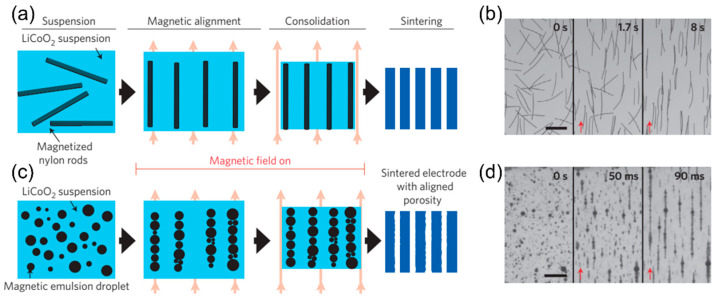
(**a**) Magnetic-field-assisted low-tortuous electrode structure using magnetized nylon rods and (**b**) magnetized nylon rods in water before and after applying the magnetic field (scale bar, 500 µm). (**c**) Magnetic-field-assisted low-tortuous electrode structure using magnetic emulsion droplets and (**d**) magnetic emulsion droplets before and after applying the magnetic field (scale bar, 75 µm). Reproduced with permission from ref. [103]. Copyright 2016, Springer Nature.

Applying magnetic force during electrode preparation is an effective means to realize highly oriented electrode structures. Either the electrode material or the decorative coating material needs to be magnetically responsive (paramagnetic or ferromagnetic) to fulfill the processing requirement. Cobalt-based cathode materials [96,97], iron oxides [98,99,100,101], cobalt oxides [102], ferrofluids [103,104], multiwalled carbon nanotubes [105], and nickel-based alloys [106] have been successfully used to produce low-tortuous electrodes. Figure 7a demonstrates that the LiCoO_2_ cathode suspension with magnetized nylon rods can align along with the magnetic field direction and can then be sintered after thermal treatment. The nylon rods decorated by the ferrofluid exhibit great alignment under the magnetic field (Figure 7b). The magnetic emulsion droplets have a similar magnetic alignment effect, which can serve as a sacrificial phase that generates straight and vertical channels after pyrolysis (Figure 7c,d). The modified LiCoO_2_ electrode with the same porosity as that of the conventional electrode can deliver a three times higher areal capacity and a high-rate capacity at 2C, indicating an effective approach without sacrificing the battery energy density [103]. An ultrahigh areal capacity (up to ≈14 mAh cm^−2^) for both the graphitic anode and LiCoO_2_ cathode adopting the same strategy has also been reported from the same group [101].

### 4.2. Electric-Field-Assisted Methods

**Figure 8:**
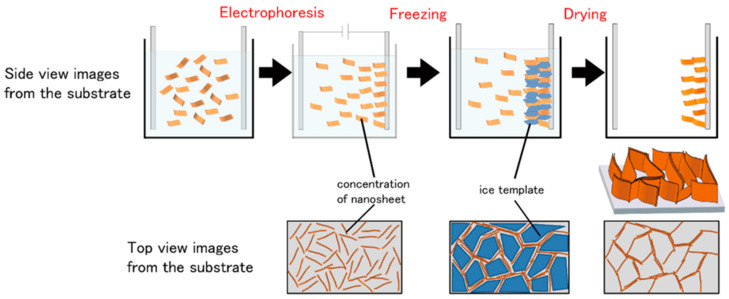
Vertically aligned film fabricated by electrophoresis and freeze drying for electrochemical capacitors. Reproduced with permission from ref. [107]. Copyright 2019, ACS Publications.

The electric field is another source of non-contact force to align the materials for low-tortuous structures. Figure 8 gives an example of combining electrophoresis and freeze drying to make a low-tortuous structure with hollow channels [107]. The electrophoresis deposition guides the graphene oxide nanosheets through the colloid to the electrode under the electric field, and the graphene oxide nanosheets can orient vertically on top of the electrode. With the following freezing process, the pores can be further expanded and secured. The vertically aligned reduced graphene can deliver a high specific capacitance (78% of that at 2 mV s^−1^) at a high scan rate of 500 mV s^−1^ in an electrochemical capacitor [107]. The authors claimed that this method can be adopted to any colloid with a negative surface charge for the film electrode preparation with a controllable thickness and pore size [107]. An earlier study also reported that sulfur-graphene composite nanosheets can self-assemble into perpendicular nanowalls driven by the electric field [108]. A good high-rate capacity (over 400 mAh g^−1^ at 8C) can be obtained in the highly oriented nanowall electrode in Li-S cells [108].

### 4.3. Shear-Field-Assisted Methods

**Figure 9:**
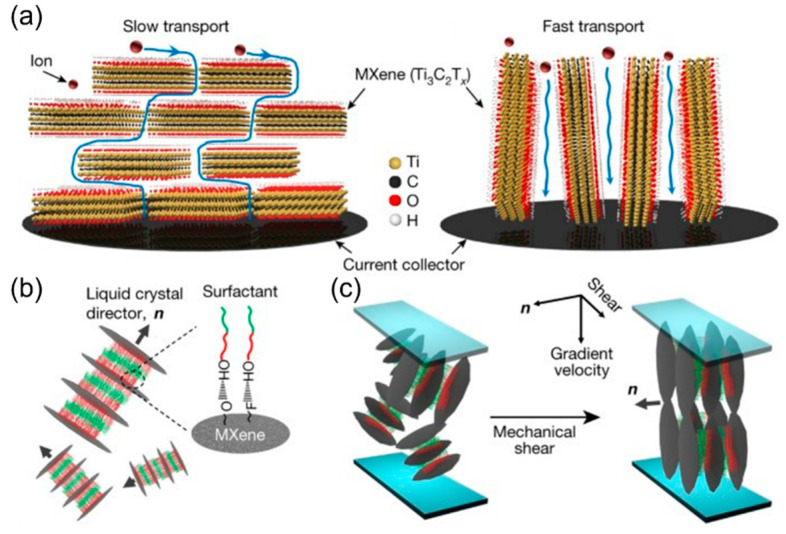
(**a**) Ion pathways in horizontally and vertically aligned MXene films. (**b**) The laminated structure of the MXene and surfactant with the hydrophilic (in red) and hydrophobic part (in green). (**c**) The shear-force-assisted vertically aligned MXene-based lamellar structure. Reproduced with permission from ref. [109]. Copyright 2018, Springer Nature.

Other than the non-contact force induced by the magnetic field or electric field, the shear force can also be applied to obtain low-tortuous structures. Yang et al. employed the mechanical shear field to directionally orientate the 2D MXene (Ti_3_C_2_T_x_) material for better ion transport, shown in Figure 9a [109]. By applying an external shear force, the Ti_3_C_2_T_x_ nanosheets can be vertically aligned even with a much greater thickness because the higher-order discotic lamellar phase formed under the assistance of a surfactant (Figure 9b) can turn perpendicularly to the shear direction (Figure 9c) [109,110,111,112]. The non-ionic surfactant used in this study (hexaethylene glycol monododecyl ether) can enhance the stacking symmetry when multiple MXene sheets integrate into liquid crystals. As a result, the areal capacitances of the vertically aligned Mxene films in supercapacitors with either a thinner or a thicker configuration (the mass loading ranging from 2.80 to 6.16 mg cm^−2^) are similar while operating at a high cycling rate (ranging from 1000 to 2000 mV s^−1^) [109]. The shear-field-assisted alignment of the liquid crystal mesophase of nanomaterials can be further utilized in other applications such as filtration, fuel cells, catalysis, and photovoltaics for superior performance [109].

## 5. Low-Tortuous Electrodes Enabled by Advanced Printing

### 5.1. Additive Manufacturing Methods

**Figure 10:**
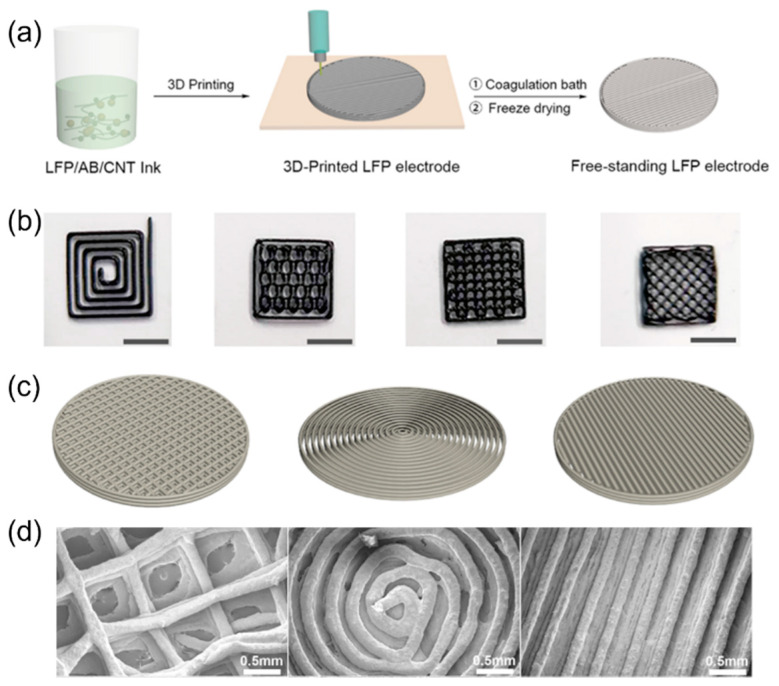
(**a**) Fabrication procedures of 3D-printed LFP electrodes. (**b**) Images of 3D-printed LFP electrodes with various patterns (scale bars: 7 mm). (**c**) Three types of patterns for 3D-printed electrodes. (**d**) Microstructure images of 3D-printed electrodes with grid, ring, and line structures. Reproduced with permission from ref. [113]. Copyright 2018, ACS Publications.

Additive manufacturing techniques, also known as 3D printing, are advanced manufacturing methods overwhelmingly adopted in multiple fields in the last decade. Large-scale customization, food, vehicles, buildings, weapons, and aerospace applications are the mainstream development directions due to the efficiency and customizable features of 3D printing. Using 3D printing to make battery components has also drawn much attention in recent years, especially in making well-aligned structures. Many electrochemical energy storage systems such as Li-ion batteries [113,114,115,116,117,118,119,120,121,122,123,124,125], Li-S/Se batteries [126,127,128,129,130,131,132], metallic lithium batteries [133,134,135,136,137], solid-state electrolytes [138], Na-ion batteries [139,140], Na-ion capacitors [141], Na-oxygen batteries [142], Ni-Fe batteries [143], Zn-ion batteries [139,144,145], and Zn-air batteries [146] have boosted their rate capability by using 3D-printed structural designs. Figure 10a illustrates how to make a freestanding electrode via 3D printing technology [113]. A viscous ink including all the necessary electrode ingredients is prepared, followed by 3D printing under a computer-controlled system to build a specific patterned structure. The electrode may need appropriate drying and/or calcination processes afterward to complete the process. Figure 10b,c demonstrate a few different patterns that can be achieved in 3D-printed electrodes. Figure 10d displays the microstructures of electrodes with various low-tortuous structures, which can facilitate high-rate battery performance. It was found that the electrode with a 3D line structure can give the highest capacity and the best cycle stability than those with the grid or ring structure, probably because of the relatively simple and consistent channel structure of the 3D line pattern [113]. The ultrathick 3D-printed electrode (8 layers; 1500 μm) gave excellent electrochemical performance, delivering a high areal capacity of 7.5 mAh cm^−2^ and a high energy density of 69.41 J cm^−2^ at a power density of 2.99 mW cm^−2^ [113].

### 5.2. Stamping Methods

**Figure 11:**
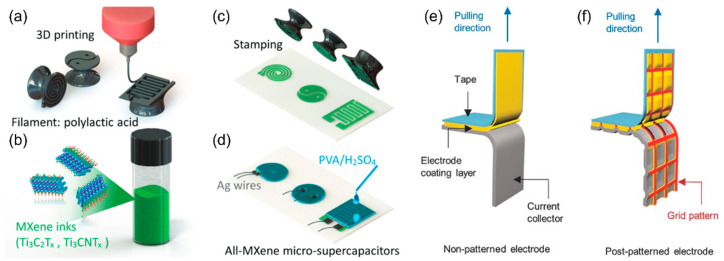
(**a**) Stamps manufactured by 3D printing. (**b**) Electrode inks of MXene for stamping. (**c**) Various electrode patterns made by stamping. (**d**) Micro-supercapacitors fabricated by stamping. Reproduced with permission from ref. [150]. Copyright 2018, Wiley-VCH. 180° peel test for (**e**) nonpatterned and (**f**) post-patterned electrodes. Reproduced with permission from ref. [154]. Copyright 2017, Wiley-VCH.

Stamping is an old-fashioned way to print repeated patterns on the substrate, which can also be used to make electrodes with special designs, particularly interdigitated structures. The interdigitated electrode design has several merits, including no separators required, reduction in ion transport resistance, and capability of being integrated into circuits and micro devices [147,148,149]. In this case, stamping is an efficient means to quickly reproduce micro-electrodes for energy storage devices. Figure 11a–d show the stamping process to fabricate interdigitated electrodes for micro-supercapacitors [150]. The stamp shapes can be facilely designed by computer-aided 3D models, followed by 3D printing using a polylactic acid filament. One study used MXene slurry as the electrode ink, and the electrode can be stamped onto the substrate with unique patterns [150]. After filling the gel electrolyte (PVA/H_2_SO_4_) and wiring silver contacts, an interdigitated solid-state micro-supercapacitor can output an areal capacitance of 50 mF cm^−2^ at 800 µA cm^−2^ [150]. The stamping methods have also been applied to prepare micro-battery electrodes with Zn//MnO_2_ [151] or self-assembled viruses [152], and graphene-based micro-supercapacitor electrodes [153]. A similar concept is utilized in a flexible node-type electrode realized by adding an imprinting step during electrode manufacturing [154]. The only difference is that the grid pattern is made by a post-roll-pressing procedure with a mesh template [154]. The post-patterned electrode also showed better adhesion on metal current collectors, measured by the test displayed in Figure 11e,f, resulting in stable electrochemical performance after 3000 flexing cycles [154].

## 6. Top-down Manufacturing Process for Low-Tortuous Electrodes

### 6.1. Laser Drilling Methods

**Figure 12:**
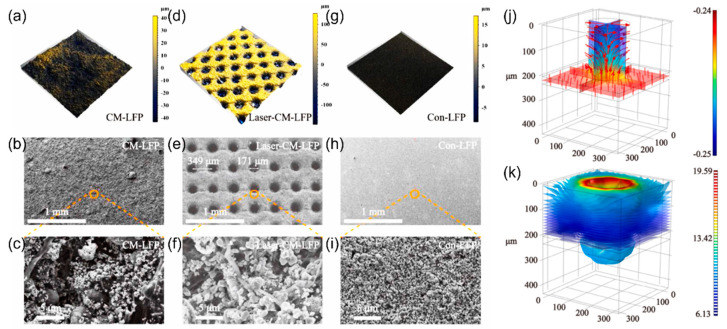
3D reconstruction images and SEM images of (**a**–**c**) CM-LFP (LFP cathode with CNFs and MWCNTs), (**d**–**f**) Laser-CM-LFP (laser-drilled CM-LFP cathode), and (**g**–**i**) Con-LFP (conventional LFP cathode). (**j**) Li-ion flux distribution, and (**k**) current distribution with laser-drilled holes in the electrode. Reproduced with permission from ref. [156]. Copyright 2021, Elsevier.

The laser has been used as a mature cutting tool for precise miniature machining on a wide variety of substrates, and the cutting focus could be 25 microns or under with high accuracy [155]. Therefore, the laser can be used to drill low-tortuous holes into electrodes to improve their high-rate capability. Figure 12a–i compare the surface morphology of the LFP electrode with multiwalled carbon nanotubes (MWCNTs) and carbon nanofibers (CNFs) (CM-LFP), the same electrode but with laser-drilled holes (Laser-CM-LFP), and the LFP electrode prepared with the traditional slurry casting process (Con-LFP) [156]. Thicker electrodes with low-tortuous holes can be fabricated with high areal capacities (3.02 and 5.33 mAh cm^−2^ from areal loadings of 20.0 and 40.0 mg cm^−2^, respectively), but still possess great cycle stability [156]. Figure 12j,k show the distribution of lithium-ion flux and current, respectively, indicating fast ion pathways in the drilled hole and concentrated current density around the edge of the hole to promote fast kinetics of electrochemical reactions [156]. The authors also pointed out that the larger the opening of the laser-drilled hole, the lower the ionic resistance that could be obtained [156]. Nevertheless, too large a pore size might degrade the mechanical property of the electrode. Many energy storage devices including Li-ion batteries [157,158,159,160,161,162,163,164,165,166], solid-state batteries [167], supercapacitors [168], and redox flow batteries [169,170] can also take advantage of laser drilling to enhance their electrochemical performance. One unique approach using a laser to create micrometer-sized through-holes is for the prelithiation of a graphite anode in the configuration of a laminated lithium-ion battery [171]. In this design, the lithium ions supplied from the additional lithium metal foil can easily penetrate multiple electrode/separator stacks in the laminated cell, shortening the prelithiation process that can be integrated into the formation cycles of the full cell [171].

### 6.2. Etching Methods

**Figure 13:**
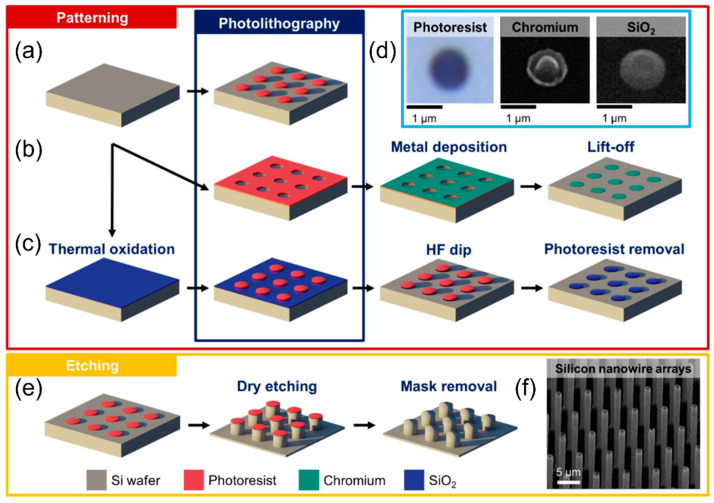
Different fabrication procedures of the vertical silicon (Si) nanowire array electrode using (**a**) photoresist, (**b**) chromium (Cr), and (**c**) silicon dioxide (SiO_2_) as patterning masks along with photolithography and multiple processing techniques. (**d**) The circular patterns made by photoresist, Cr, and SiO_2_ masks. (**e**) ICP/RIE process to etch silicon for the low-tortuous array structure. (**f**) SEM image of the vertical Si nanowire arrays. Reproduced with permission from ref. [181]. Copyright 2021, Springer Nature.

Etching is a classic method to remove materials from the substrate by using corrosive, caustic, or abrasive substances, mostly utilized for printing in ancient times. In modern times, etching has been widely used in advanced semiconductor manufacturing, and many different etching technologies have been developed [172,173,174,175]. Battery scientists have also adopted etching techniques including inductively coupled plasma/reactive ion etching (ICP/RIE) [176,177,178,179,180,181,182], catalytic etching [183], and wet etching [184,185,186] to make different 3D electrode structures for better high-rate performance. Dry etching is a powerful process that can accurately remove unwanted materials with exact quantity and shape with the assistance of patterning and photolithography (Figure 13). The array patterns can be obtained by photolithography (Figure 13a), combining with metal deposition and lift-off (Figure 13b) or with pre-oxidation, acid etching, and photoresist removal processes (Figure 13c) [181]. The low-tortuous silicon electrode forms in vertical arrays after ICP/RIE etching and mask removal (Figure 13e,f), and a high aspect ratio of up to 22 is achievable [181]. Another etching-enabled low-tortuous design is that Duan et al. developed a hierarchically porous holey-graphene framework structure by a simple wet-etching process using hydrogen peroxide (H_2_O_2_), promoting high-rate capability even at a rate as high as 10C [186]. The 3D porous electrode structure facilitates rapid ion transport by offering internally straight channels in the holey-graphene composite material [186]. The cost-ineffectiveness of dry etching and the poor structural uniformity caused by wet etching are potential limiting factors for etching processes to be employed in energy storage applications.

### 6.3. Cutting Methods

**Figure 14:**
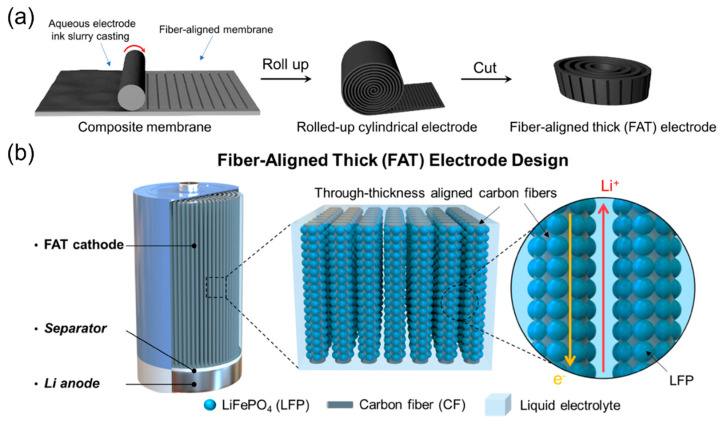
(**a**) Fabrication process of the FAT electrode. (**b**) Design concept of the FAT electrode. Reproduced with permission from ref. [187]. Copyright 2020, ACS Publications.

Cutting or chopping is another method to accomplish top-down manufacturing of low-tortuous electrodes. Fu et al. designed a fiber-aligned thick (FAT) electrode by simply rolling up the electrode with a fibrous substrate followed by cutting into a spiral structure perpendicular to the axial direction (Figure 14a) [187]. The FAT electrode not only has a high areal capacity, controlled by the cutting thickness, but also possesses through-thickness electrode alignment and ample electrolyte channels, facilitating both high energy density and high power density [187]. The fabricated 1 mm thick electrode is made with high areal loading of 128 mg cm^−2^, which can still deliver a high capacity of 155 mAh g^−1^ under 0.5 mA cm^2^ [187]. Other groups also adopted a similar strategy to cut electrode rolls into low-tortuous electrodes for the applications in lithium-oxygen batteries [188] and metallic lithium anodes [189]. Unlike winding, graphene nanosheet composites can form a thick horizontally aligned monolith after vacuum filtration and compression, which can be cut and rotated to create vertically aligned channels as low-tortuous electrodes as well [190]. To cut stacked electrodes perpendicular to the alignment direction may require special tools and crafts to obtain electrodes with a consistent thickness.

## 7. Summary and Outlook

**Figure 15:**
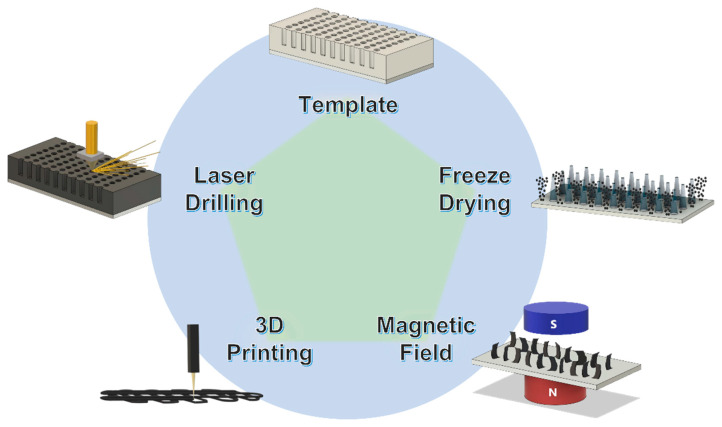
The mainstream processes of low-tortuous electrode manufacturing.

In the past decade, a tremendous number of smart manufacturing methods have been developed to make low-tortuous electrodes and solid-state electrolytes. Most of them share the same features including high power density, high energy density, high areal loading, and highly efficient active material utilization. Figure 15 summarizes the most popular processes to produce low-tortuous structures. Templating is a classic manufacturing technique that uses a sacrificial framework to form the structure with the same 3D morphology, which gives excellent product uniformity and structural controllability. Freeze drying is a mature drying process implemented when heating is unfavorable to the material. The frozen solvent (usually ice) can grow directionally along with the cooling gradient, leading to vertical channels after the ice crystals are removed during sublimation. Magnetic-field-assisted low-tortuous structure formation is a quite creative approach, which employs the nature of the magnetic moment induced by the applied magnetic field, offering the necessary torque for good alignment. Three-dimensional printing is an emerging technology disrupting various industries. Scientists and engineers make use of the additive manufacturing to produce electrodes with several special state-of-the-art structures for different kinds of applications, including a highly oriented electrode to improve high-rate performance. Laser drilling can precisely control the shape, density, size, and depth of the holes on electrodes, resulting in the design flexibility of making porous electrodes or substrates.

This review article briefly introduces various smart manufacturing methods for low-tortuous structures, which could be implemented in other advanced applications in addition to electrochemical energy storage devices. Manufacturing cost has always been the most fundamental determinant of mass production and commercialization. The cost of the template material and the process of template removal must be low enough for large-scale operation. Bio-derived templates may encounter difficulties with varying quality from different batches of organic matters, although the sustainability of biologic materials is still undoubtedly attractive. Freeze drying seems to be a cost-effective method to build a low-tortuous structure, but the dimension and morphology of each ice crystal column might not be identical, requiring in-depth research to enhance uniformity. Magnetic-field-assisted vertically aligned structure formation needs magnetic substances, which increases the inactive material loading and reduces the versatility of this method. Three-dimensional printing should be further improved by minimizing the size of printed products (i.e., added resolution), so the electrode design will not be limited. Laser drilling is a promising non-contact manufacturing technique due to its high precision and speed. However, the power consumption and facility cost of laser devices could be high but is yet to be confirmed for the production cost-effectiveness before scaling up. To sum up, there are plenty of approaches to construct hierarchical low-tortuous electrodes, but the structural uniformity, low material and processing cost, and device performance targets must be met simultaneously using the same manufacturing technology to achieve the ultimate goal for high-rate energy storage systems.

## Data Availability

All collected data are presented in the manuscript.
